# A Pitfall in Heavy Metal Separation with Amino‐modified Silica Adsorbents from Aqueous Solution: The Occurring pH Shift

**DOI:** 10.1002/open.202200034

**Published:** 2022-03-10

**Authors:** Friederike Kriese, Stephan Lassen, Helena Horn

**Affiliations:** ^1^ Institute of Thermodynamics Helmut-Schmidt-University / University of the Bundeswehr Hamburg Holstenhofweg 85 22043 Hamburg Germany

**Keywords:** adsorption, amino-functionalization, heavy metal, nickel, cobalt

## Abstract

Selective separation of heavy metal ions from acidic aqueous solutions is of strong interest for certain industrial processes, such as electroplating, as well as environmental protection, for example battery recycling. Amino‐functionalized adsorbents are often discussed as suitable material for this purpose. Herein, two silica‐based adsorbents functionalized with 3‐aminopropyl‐ and 3‐[2‐[2‐aminoethylamino]‐ethylamino]‐propyl‐ligands resulting in adsorbents MonoA and TriA, respectively, were investigated regarding their separation behavior with focus on nickel(II) and cobalt(II) in batch as well as continuous flow experiments in acidic aqueous solutions. For both adsorbents, pH shifts into the alkaline range were observed in the process solutions, causing precipitation of metal hydroxides mainly in the particle pores in case of adsorbent MonoA and a combination of precipitation and adsorption regarding adsorbent TriA.

Contrary to prior studies, our findings evidence that amino‐functionalized adsorbents are not applicable for nickel(II) and cobalt(II) in selective adsorption processes and additionally demonstrate that, besides batch investigations, continuous flow experiments are essential for well‐founded adsorbent selections in process development.

## Graphical abstract

**Unspecific separation of heavy metal ions** such as cobalt(II) and nickel(II) on amino‐functionalized adsorbents has been found due to severe pH shifts into the basic milieu caused by metal hydroxide precipitation instead of specific metal complexing. These findings contradict reports on such adsorptive processes in literature and prove that continuous flow examinations are necessary for a sufficient adsorbent selection.

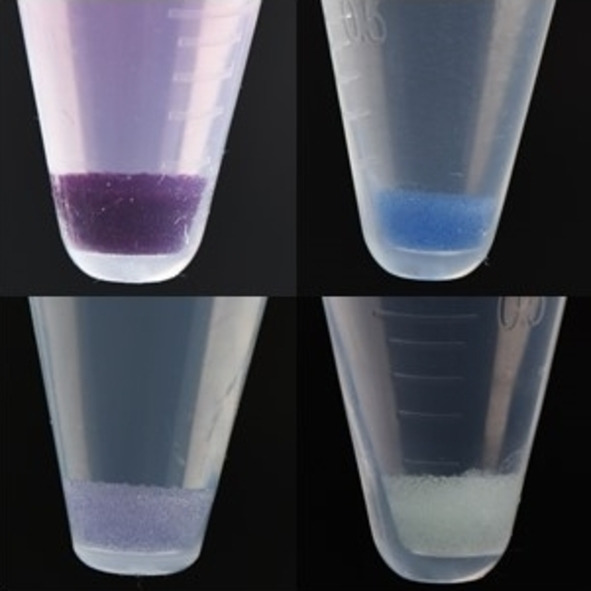

## Introduction

Worldwide, heavy metals such as cadmium (Cd), chromium (Cr), cobalt (Co), lead (Pb), and nickel (Ni) are frequently detected in industrial effluents,[^1^, ^2^, ^3^] through which these hazardous metals can be extensively introduced into the environment.[^4^, ^5^]

It is well known that such industrial released pollutants pose a serious threat to ecosystem and human health.[^6^, ^7^, ^8^] Due to their non‐degradability and toxicity, heavy metals bio‐accumulate in marine and terrestrial food webs[^9^, ^10^] and finally in organs of the human body, where they are able to cause severe health damage.[^11^, ^12^] Therefore, elimination of toxic heavy metals from contaminated industrial sewage flows represents an important task of health protection. Apart from that, sustainable recovery of certain heavy metals, for example nickel(II) and cobalt(II), from industrial process or waste water could be essential, since they are key elements in the context of prospective fossil‐free energy generation and mobility.[^13^, ^14^, ^15^]

A widely used elimination method for nickel(II) or cobalt(II) in wastewater treatment is adsorption because of high separation capacities and good regeneration capabilities of the various applied adsorbents such as activated carbons, clays (e. g., bentonite), zeolites, biopolymers (e. g., chitosan), and minerals (e. g., hydroxyapatite).[^16^, ^17^] Over the past years, the selective removal of heavy metals, including nickel(II) or cobalt(II), from aqueous solutions using amino‐functionalized silica has been investigated in several studies.[^18^, ^19^, ^20^, ^21^, ^22^] In these publications, the individual adsorption capacities of the therein investigated amino‐modified adsorbents were determined by means of batch adsorption experiments at various initial pH values in the sample solutions. For this purpose, different start values were adjusted in a pH range from 1.5 to 6.0, but interestingly not controlled under equilibrium conditions at the end, although the basic character of amino‐ligands was demonstrated by detected positively charged surfaces of the functionalized adsorbents in acidic milieus.^[23]^

We present systematic investigations of the adsorption performance of two amino‐functionalized silica adsorbents with regard to nickel(II) and cobalt(II) in a continuous (flow) and discontinuous (batch) experimental mode as a function of the pH shift monitored continuously over time and after reaching the equilibrium state, respectively. Our results verify drastic pH shifts into the basic range in both modes, regardless of the initially set pH values (2.2, 3.5, 6.0) in the sample solutions. Hence, we evidence that the observed heavy metal separations cannot solely be based on an adsorption mechanism. Instead, they are primarily caused by precipitation of metal hydroxides in the surrounding liquid phase and to an increased extent inside the adsorbent pores.

## Results and Discussion

A mesoporous and spherical silica gel was utilized as support matrix of both investigated adsorbents and functionalized by silanisation with 3‐aminopropyltriethoxysilane (adsorbent MonoA) and 3‐[2‐[2‐aminoethylamino]‐ethylamino]‐propyltrimethoxysilane (adsorbent TriA), respectively. Nitrogen mass fractions of 1.34 % and 3.57 % for MonoA and TriA were determined by elemental analysis, reflecting surface coverages of the adsorbents with ligands of 954 μmol_Ligand_ g^−1^_Adsorbent_ and 850 μmol_Ligand_ g^−1^_Adsorbent_ for MonoA and TriA, respectively.

### Batch Mode Experiments

**Figure 1:**
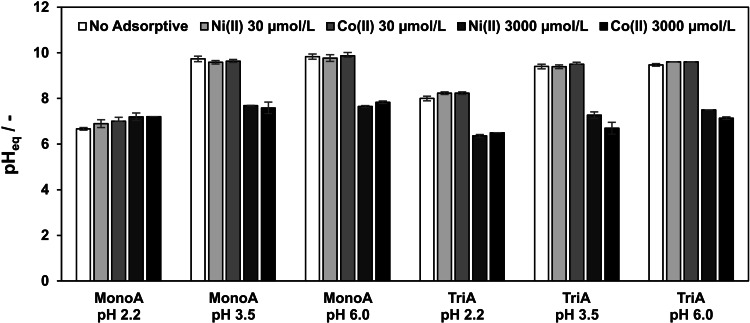
pH value in equilibrium state (pH_eq_) depending on adsorptive (nickel(II), cobalt(II)), initial adsorptive concentration (0, 30 or 3000 μmol L^−1^), adsorbent (MonoA, TriA) and initial pH value in batch adsorption experiments (T=20 °C).

Discontinuous separation investigations of the adsorbents (m=50 mg) in pure aqueous solutions (V=5 mL) with different initial pH values of pH 2.2, 3.5 and 6.0 showed extreme pH shifts into a neutral to basic aqueous milieu in the equilibrium state (pH_eq_=6.7 to 9.8) after 24 hours contact time (Figure 1, each first bar “No Adsorptive”). Based on the assumption that the p*K*_b_‐values of the amino groups of both adsorbents MonA and TriA are comparable to the values of the organic bases propylamine (p*K*_b_=3.1) and 1‐*N*‐methyldiethylenetriamine (p*K*_b,1_=4.1; p*K*_b,2_=4.8; p*K*_b,3_=10.7), it can be assumed that here the amino groups of the immobilized ligands, except the presumed weakly basic one of TriA, reacted according to Equation (1) in aqueous solution, causing the observed pH shifts:[^24^, ^25^]

(1) $$R\text{-}\mathrm{NH}{}_{2}+H{}_{2}O↔R\text{-}\mathrm{NH}{}_{3}{}^{+}+\mathrm{OH}{}^{-}$$

Considering the theoretical amount of potentially reacting amino groups (approximately 48 μmol for MonoA and 85 μmol for TriA related to 50 mg of adsorbent applied in the experiments), calculations predict theoretical values of around pH_eq_=12 in the experimental set‐ups. Taking into account that the reaction described in Equation (1) basically proceeds inside the pore volume (approx. 3.8×10^−2^ mL), the deviation to the measured maximum pH_eq_=9.8 (Figure 1, “No adsorptive”) indicates that the pH_eq_ value near the adsorbents inner surface might be higher than in the surrounding liquid phase. Even in presence of nickel(II) and cobalt(II) as adsorptives at low and high concentrations (30 and 3000 μmol L^−1^), both adsorbents induced increased values of pH_eq_ between 6.4 and 9.9 (Figure 1).

**Figure 2:**
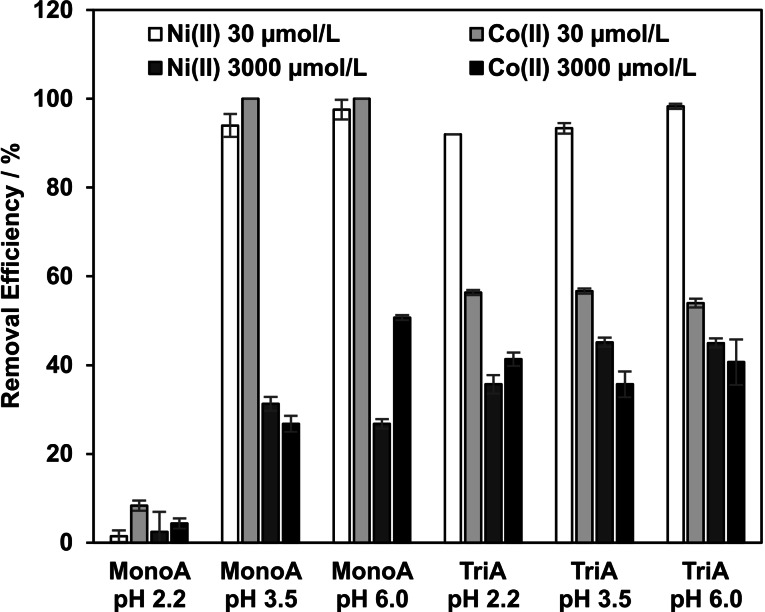
Removal Efficiency in equilibrium state depending on adsorptive (nickel(II), cobalt(II)), initial adsorptive concentration (30 or 3000 μmol L^−1^), adsorbent (MonoA, TriA) and initial pH value in the batch adsorption experiments (T=20 °C).

In batch experiments with MonoA at initial pH values of pH=3.5 and pH=6.0 and adsorptive concentration of 30 μmol L^−1^ for nickel(II) and cobalt(II), respectively, the resulting maximum pH shift (pH_eq_=9.9) was similar to that found from analogous experiments with no adsorptive. Removal Efficiencies (RE) of 90 to 100 % were obtained for both adsorptives (Figure 2). At high adsorptive concentrations (3000 μmol L^−1^), the value of pH_eq_ for MonoA increased only to 7.8 at maximum (Figure 1) and RE of 30 to 50 % were observed (Figure 2).

In an alkaline milieu of pH=9.0, the saturation concentration of soluble nickel(II) and cobalt(II) is 6 μmol L^−1^ and 12 μmol L^−1^, respectively, before metal hydroxide precipitation [Eq. (2)] takes place.^[26]^

(2) $$M{}^{\mathrm{II}}+2\;\mathrm{OH}{}^{-}↔M\left(\mathrm{OH}\right){}_{2}$$

As assumption 1, it can be hypothesized that the separation mechanism is dominated by metal hydroxide precipitation because the saturation concentrations of metal(II) ions are exceeded. Contrary to earlier studies,[^19^, ^20^, ^27^, ^28^, ^29^, ^30^] which described separation by complexation between amino groups of 3‐aminopropyl‐ligands and metal ions, the detected lower pH shifts at 3000 μmol L^−1^ (Figure 1) could be a consequence of the discussed metal hydroxide precipitation, because the concentration of hydroxide ions is decreased according to Equation (2).

As assumption 2, a higher amount of positively charged metal(II) ions competes with water molecules or oxonium (H_3_O^+^) ions for interaction with the ligands’ amino groups, leading to an increased inhibition of the protonation reaction [Eq. (1)] and consequently to a lowered OH^−^ formation.

**Figure 3:**
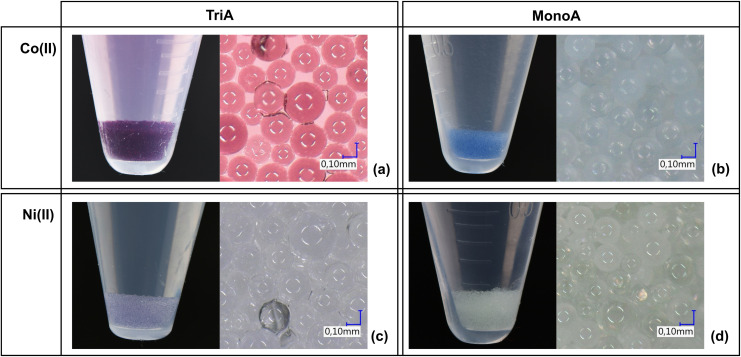
Colored adsorbents after batch separation experiments in equilibrium state. Separation of cobalt(II) by TriA (a) and cobalt(II) by MonoA (b). Separation of nickel(II) by TriA (c) and nickel(II) by MonoA (d). Macroscopic representation (left) and in 100x magnification (right).

Especially at the investigated high adsorptive concentrations, different colorations of the adsorbent MonoA were noticed after contact with the corresponding solutions (Figure 3). In case of nickel(II), the MonoA particles took on a light green color and additionally a greenish precipitate was formed on the surface of the adsorbent bulk (Figure 3d). This observation supports assumption 1, since Ni(OH)_2_ precipitates as green, voluminous sediment under basic conditions.^[31]^

Cobalt(II) immediately caused an intense blue coloration of the adsorbent MonoA and pale pink streaks aggregated on the surface of the adsorbent bulk in equilibrium state (Figure 3b). This latter observation is another evidence for metal hydroxide formation, here pink Co(OH)_2_, while the blue color indicates a formation of soluble blue tetrahydroxo ([Co(OH)_4_]^2−^) or hexahydroxo ([Co(OH)_6_]^4−^) cobaltate ions.^[31]^ The observed accumulation of cobaltate anions in the adsorbent bulk is caused by interaction with the protonated amino groups on the surface of MonoA.

In the adsorption experiments with MonoA at an initial pH value of 2.2, hardly no removal of nickel(II) and cobalt(II) was observed (Figure 2, first set of bars). Under these conditions, the values of pH_eq_ shifted only to a weakly acidic (pH_eq_=6.7) or neutral milieu (pH_eq_=7.0) (Figure 1, first set of bars), in which a hydroxide precipitation can be excluded.^[26]^ The majority of all formed hydroxide ions are converted by the relatively high amount of H_3_O^+^ ions into water molecules.

Compared to analogous experimental set‐ups with no adsorptive, adsorbent TriA (3000 μmol L^−1^ cobalt(II) / nickel(II); pH=3.5 or pH=6.0) caused lower pH shifts to maximum pH_eq_ values of 7.5 and 7.1 for nickel(II) and cobalt(II), respectively. In contrast to MonoA, the pH shifted rather to a neutral milieu than to a weakly basic one (Figure 1). RE for TriA of about 36 % for nickel(II) and 41 % for cobalt(II) were observed for the high heavy metal ion concentrations in difference to MonoA, when the initial pH value was set to 2.2.

As hypothesized in assumption 2, these results indicate that the metal(II) ions preferentially form metal(II) complexes with protonated triamine ligands ([H_2_TriA]^2+^) on the adsorbent surface to form [M(TriA)]^2+^ and [M(TriA)_2_]^2+^ complexes according to Prue and Schwarzenbach (1950), who investigated several heavy metal complexes with diethylentriamine in the aqueous phase, including those which are formed with nickel(II) and cobalt(II).^[32]^

Different colorations of TriA were noticed after contact with the different adsorptive solutions (3000 μmol L^−1^) as well. In the batch experiment with the adsorbent TriA and nickel(II), a violet colored adsorbent (Figure 3c) was generated. The color is attributable to the formation of organic nickel(II) complexes between the hydrated nickel(II) (coordination number 6) and the tridentate triamine ligands. Previous spectrometric experiments with diethylenetriamine (dien) in aqueous solutions of nickel(II)nitrate at different molecular ratios evidenced the formation of blue violet complex ions of the compositions [Ni(dien)]^2+^ (absorption maximum λ_max_=580 nm) and [Ni(dien)_2_]^2+^ (λ_max_=540 nm).[^33^, ^34^] Scheglova and Popova (2020) studied mixed‐ligand complexes of nickel(II) and copper(II) in aqueous solution and demonstrated comparable maximum absorption wavelengths for the homoleptic nickel(II)‐dien complexes of 560 nm ([Ni(dien)_2_]^2+^) and 610 nm ([Ni(dien)]^2+^).^[35]^ Their reported optimum pH range of 6.2 to 7.5 for the generation of [Ni(dien)]^2+^ complex ions matches our determined pH_eq_ range of 6.4 to 7.5 in the batch experiments for nickel(II) separation on TriA (Figure 1). Consequently, the adsorption mechanism mainly relies on complexation of one nickel(II) ion by one triamine ligand to a [Ni(TriA)]^2+^ complex, irrespective of the initial pH values of the analyzed solutions. The resulting color of TriA in the equilibrium state was purple (Figure 3a) when adding cobalt(II) to the sample solutions. Comuzzi et al. (2001) measured the absorption spectra of different cobalt(II)‐dien complex ions ([Co(dien)]^2+^, Co(dien)_2_]^2+^) in dimethylsulfoxide (DMSO)^[36]^ and determined absorption maxima of λ_max_=510 nm in the green, and of λ_max_=470 nm in the blue color range. Thus, the transmitted light of the analyzed [Co(dien)]^2+^‐ and [Co(dien)_2_]^2+^‐DMSO solutions should be purple and yellow, respectively. By contrast, Whealy and Colgate (1956) suggested a structure of [Co(dien)]^2+^ for the yellow complex in aqueous solution.^[34]^ Own laboratory experiments (data not shown) in aqueous solution with a cobalt(II)/dien molecular ratio of 1 : 1 and with an excess of dien (1 : 5) resulted in purple and yellow‐colored solutions, respectively, which agrees well with the results of Comuzzi et al.

Consequently, the observed colorations of the adsorbent TriA confirm that the separation mechanism of cobalt(II) with TriA in aqueous solution, likewise to nickel(II), also based mainly on metal complexing of one cobalt(II) with one TriA ligand. Hydroxide precipitations were not observable in both cases. However, the observed pH shifts, especially at low concentrations (30 μmol L^−1^, pH_eq_=8.2 to pH_eq_=9.6, Figure 1), indicate a formation of hydroxide ions and a subsequent metal precipitation.

### Continuous Mode Experiments

**Figure 4:**
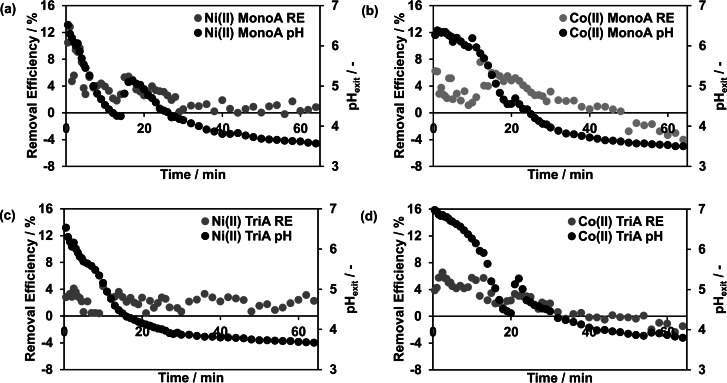
Removal Efficiency and exiting pH value as a function of time (continuous mode). Initial metal(II) ion concentration 3000 μmol L^−1^ and pH=3.5 (T=20 °C). Separation of nickel(II) (a) and cobalt(II) (b) by MonoA. Separation of nickel(II) (c) and cobalt(II) (d) by TriA.

As in the batch experiments, the amount of formed hydroxide ions is limited by the mass of the applied adsorbent in the subsequent continuous flow experiments, whereas H_3_O^+^ ions are available in excess quantities due to the continuously supplied acidic process solutions. In order to study the impact of a continuous acidic feed, flow experiments were conducted with MonoA and TriA in packed beds of 0.1 g and nickel(II) as well as cobalt(II) in the feed solutions (initial pH=3.5, 3000 μmol L^−1^). Similar to the batch mode experiments, the pH values shifted strongly towards values between 6.0 (weakly acidic) to 7.0 (neutral) in the first minutes of all continuous experiments with both adsorbents (Figure 4; Supplement Figure S1). This indicates a preferred protonation of the ligand amino groups [Eq. (1)], which caused the formation of a high amount of hydroxide ions, mostly inside the porous structure of the adsorbent particle, that reacted with the relatively low amount of H_3_O^+^ ions entering the column. Consequently, the reaction of hydroxide ions with H_3_O^+^ ions was minor so a high amount of hydroxide ions most likely existed inside the pores at the beginning of the flow experiments.

During the investigation of MonoA with nickel(II) as adsorptive in the continuous mode, RE per volume fraction between 2 to 13 % were observed in the first 30 min of process time (Figure 4a).

In the experimental set‐up with MonoA and cobalt(II), lower RE between 1 to 8 % per volume fraction were determined over a time interval of 48 min (Figure 4b). After 48 min, the pH value of the exiting solution approached the initial pH value of 3.5 (Figure 4b), showing that the limited amount of hydroxide ions was nearly converted. Further proceeding of the experiment at the originally purposed process pH value (pH=3.5) led to a release of already separated cobalt(II) resulting in negative RE. That is, Co(OH)_2_ and [Co(OH)_4_]^2−^ ions dissolve to hydrated [Co(H_2_O)_6_]^2+^ ions in acidic milieus which caused the observed increase of cobalt(II) concentration in the collected fractions [Eq. (2)].[^31^, ^37^]

**Figure 5:**
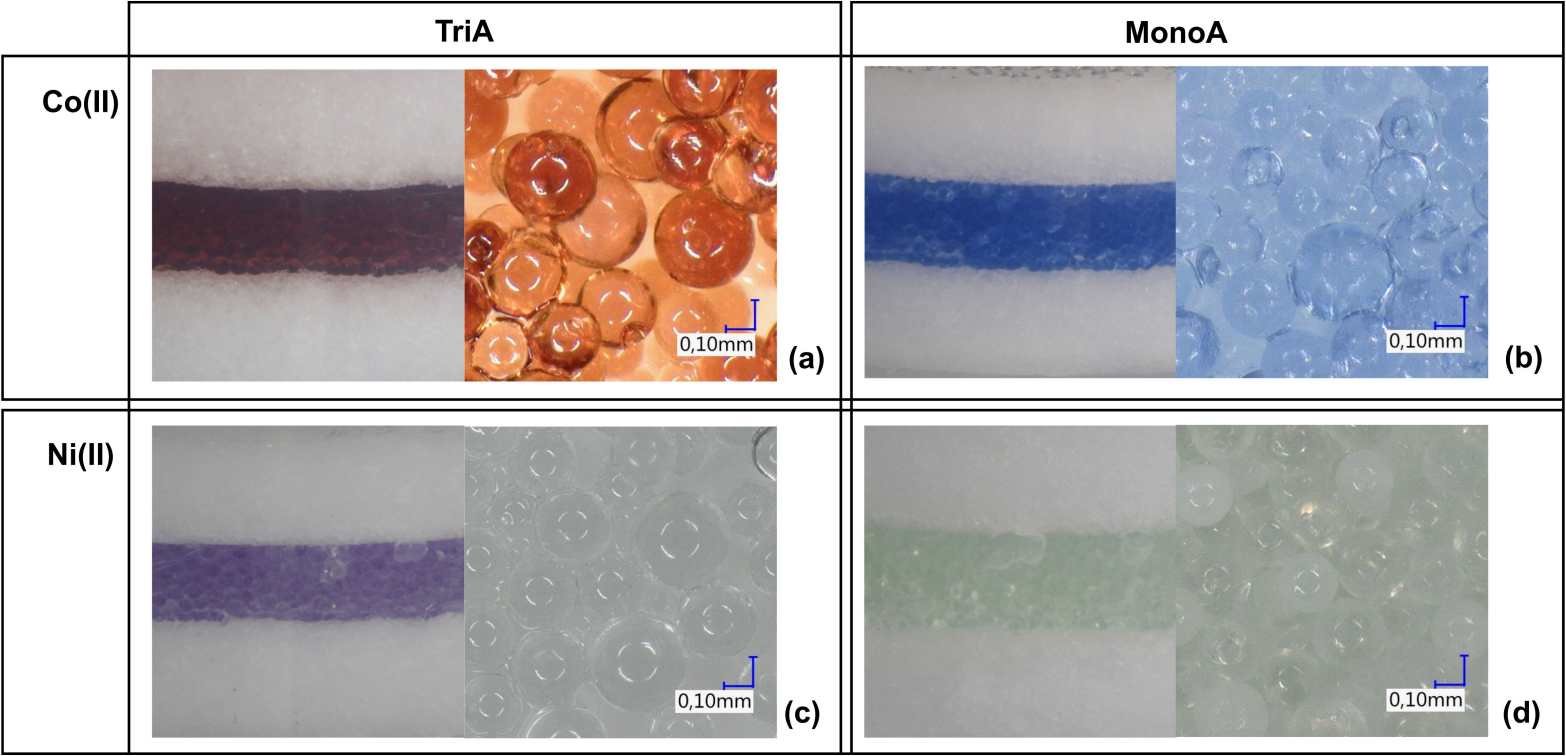
Colored adsorbents after continuous separation experiments. Separation of cobalt(II) by TriA (a) and cobalt(II) by MonoA (b). Separation of nickel(II) by TriA (c) and nickel(II) by MonoA (d). Macroscopic representation (left) and in 100x magnification (right).

The observed green coloration of the adsorbent bed (Figure 5d) in the experimental set‐up nickel(II)/MonoA was similar to the adsorbent color in batch mode due to Ni(OH)_2_ precipitation inside the pores. When employing cobalt(II), the MonoA fixed bed showed a blue coloration in the first 5 min of processing (Figure 5b). According to the batch mode experiments, this change of color also indicates the formation of cobaltate ions. After 20 min of process time, a gradual adsorbent decoloration emerged but the separation of cobalt(II) was still noticeable in the following volume fractions (Figure 4b), which supposes that the cobaltates were converted into Co(OH)_2_ precipitate in the adsorbent pores by replacement of two or four hydroxide ions out of the cobaltate complex by surplus H_3_O^+^ ions.

In the case of TriA, the experimental set‐up with nickel(II) yielded a total separation of 20 μmol over the whole process time (Figure 4c). In the experiment with TriA and cobalt(II), also 20 μmol of the metal(II) ions were separated. Negative RE were determined after 34 min of process time at pH=4.3, proving that cobalt(II) dissolved again from the adsorbent (Figure 4d).

Continuous adsorption experiments with TriA and nickel(II) (Figure 5c) as well as cobalt(II) (Figure 5a) generated a violet and an orange‐yellow coloration of the adsorbent, respectively, which implies that the effect of metal complexing took place, too. The observed orange‐yellow color of TriA compared to that in the batch experiments (purple) might be caused by a combination of purple [Co(TriA)]^2+^ and yellow [Co(TriA)_2_]^2+^ complexes, which are likely formed under these non‐equilibrium conditions.

Over time, the cobalt(II)‐TriA complexes got instable with the successively declining pH value (Figure 4), due to a stepwise complexe dissociation and subsequently to a release of cobalt(II) into the process solution. Shinora et al. (1977) investigated the ligand dissociation of cobalt(II)‐dien complexes and showed that with increasing H_3_O^+^ ion concentration their dissociation rate constants increase to a limited value (k_a_/s^−1^) as well. Interestingly, the [Co(dien)_2_]^2+^ complex (k_a_=1.8×10^4^ s^−1^) dissociates 20 times faster than the [Co(dien)]^2+^ complex (k_a_=8.7×10^2^ s^−1^),^[38]^ which is also reflected in the different complex formation constants of lg(*K*_[Co(dien)2]2+_)=6.0 and lg(*K*_[Co(dien)]2+_)=8.0.^[32]^ The fact that the nickel(ll)‐TriA complexes stayed stable under the experimental conditions could be caused by the higher complex formation constants for nickel(ll)‐dien complexes (lg(*K*_[Ni(dien)2]2+_)=8.2, lg(*K*_[Ni(dien)]2+_)=10.7).^[32]^

## Conclusion

Our experimental results proved that both amino‐functionalized silica caused a severe pH shift in the analyzed aqueous solutions and that the separation process with adsorbent MonoA was dominated by an unspecific metal hydroxide precipitation. These findings contradict earlier studies, which discussed a specific adsorption by metal complexation between amino groups of the applied adsorbents and various heavy metal ions under acidic conditions.[^19^, ^20^, ^27^, ^28^, ^29^, ^30^] An inhibition of adsorption by amine group protonation was only addressed for pH values <3.^[28]^ In contrast, adsorbent TriA formed metal(II) complexes at higher pH values. In this case, the separation process can be defined as adsorption. However, at low adsorptive concentrations, interfering unspecific precipitation effects were determined due to the high amount of hydroxide ions in the system. Equivalent experiments in the continuous mode confirmed these results as well. Therefore, we state that amino‐modified silica adsorbents are unsuitable for selective heavy metal separation processes. This statement is underlined by the reversion of the initially observed precipitation and adsorption, respectively, along with the determined pH shifts over the process time.

## Experimental Section

### Synthesis of Amino‐functionalized Silica Gels

Silica gel SiliaSphere™ PC, (particle size: 200–500 μm; average pore diameter: 100 Å; pore volume: 0.75 mL g^−1^; average surface area: 330 m^2^ g^−1^) from Silicycle (Quebec City, Canada) was utilized as adsorbent matrix. The two functionalized adsorbents (MonoA, TriA) were synthesized by silanisation with 3‐aminopropyltriethoxysilane (purity 98 %) and 3‐[2‐[2‐aminoethylamino]‐ethylamino]‐propyltrimethoxysilane (purity 85 %), both from abcr (Karlsruhe, Germany), according to Braaß et al., Thiesen et al., Helmholz et al., and Kötke.[^39^, ^40^, ^41^, ^42^] Depart from the described reaction conditions, the temperature as well as the time were raised to 100 °C and 4 hours, respectively. Instead of toluene, xylene was applied as solvent.

#### Characterization of Functionalized Silica Gels by Elemental Analysis

The amount of functionalized ligands on the modified silica surface was quantified by elemental analysis via the carbon and nitrogen content employing a Vario EL cube (Elementar Analysensysteme, Langenselbold, Germany) with helium as carrier gas. Sulphanilic acid (p.a., Merck, Darmstadt, Germany) was used as calibration standard. Each sample consisted of 10 mg adsorbent and was analyzed in triplicate.

### Quantification of Heavy Metals by ICP‐MS

The determination of nickel(II) and cobalt(II) concentrations in aqueous samples was conducted by Inductively Coupled Plasma Mass Spectrometry (ICP‐MS) with a 7800 ICP instrument from Agilent Technologies (Waldbronn, Germany). For system calibration, stock solutions with nickel(II) and cobalt(II) concentrations of 100 ppm were prepared with Ni(NO_3_)_2_ ⋅ (H_2_O)_6_ (purity 99 %) from Carl Roth (Karlsruhe, Germany) and Co(NO_3_)_2_ ⋅ (H_2_O)_6_ (purity >98 %) from Merck (Darmstadt, Germany). Standard solutions in varying concentrations were produced from the stock solutions by dilution with a mixture of 1 wt% of nitric acid (65 wt%) and ultrapure water.

### Microscopic Analysis of Adsorption Material

Adsorbents were optically analyzed after batch as well as continuous experiments with a digital light microscope (VHX‐6000, Keyence, Osaka, Japan) and documented in 100× magnification. The adsorbent specimens were prepared on microscope slides with single cavity (BMS Microscopes, Capelle a. d. Ijssel, Netherlands) directly from the original sample solutions.

#### Batch Adsorption Experiments

Batch adsorption experiments with 50 mg of functionalized adsorbent (MonoA, TriA) in 5 mL sample solution were executed in 15 mL centrifuge tubes from VWR International (Darmstadt, Germany). Sample solutions were produced by dilution of corresponding working solutions (30000 μmol L^−1^), which were prepared with Ni(NO_3_)_2_ ⋅ (H_2_O)_6_ (purity 99 %) from Carl Roth (Karlsruhe, Germany) and Co(NO_3_)_2_ ⋅ (H_2_O)_6_ (purity >98 %) from Merck (Darmstadt, Germany). The concentrations of heavy metal ions in the sample solutions varied between 0, 30 and 3000 μmol L^−1^. Each solution's pH value was determined with an SI Analytics Lab 875 pH meter (Xylem, New York, NY, USA) and adjusted to 2.2; 3.5 or 6.0 with 65 wt% nitric acid (Suprapur®, Merck, Darmstadt, Germany). Typical industrial process conditions were represented by an initial pH value of 3.5, whereas the pH value of 2.2 was selected in order to avoid metal hydroxide precipitation.^[26]^ Ultrapure water of pH=6.0 was taken as reference. In order to achieve equilibrium conditions, the centrifuge tubes were rotated in an overhead rotator (Sunlab, Mannheim, Germany) for 24 h at 40 rpm and 20 °C. Subsequently, the pH_eq_ values were determined and aliquots of each sample were taken for ICP‐MS analysis. The Removal Efficiency (RE*)* was calculated as the ratio between the adsorbed amount of metal(II) ions *n*_ad_ and the total initial amount of metal(II) ions *n*_initial_ in the liquid phase:

(3) $$\mathrm{RE}=(n{}_{\mathrm{ad}}/n{}_{\mathrm{initial}})\;\cdot \;100\;\%$$

#### Continuous Adsorption Experiments

Sample solutions (500 mL) were prepared as described above with nickel(II) or cobalt(II) (3000 μmol L^−1^) and without metal ions, respectively, and the pH values adjusted to 3.5 with 65 wt% nitric acid. All experiments were carried out in 8 mL‐Chromabond Flash columns (Macherey‐Nagel, Düren, Germany). Therefore, an amount of 0.1 g adsorbent (MonoA or TriA) was fixed by two frits (polypropylene) inside the column. Before the experiment started, the adsorbent material was wetted with ethanol (p.a., Merck, Darmstadt, Germany). Subsequently, the column was filled with sample solution and connected to a HPLC‐pump (Azura P4.1S, Knauer, Berlin, Germany). At a flow rate of 5 mL min^−1^ varying volume fractions were collected according to the following intervals: 30 s for 0 to 6 min, 60 s for 6 to 30 min and 120 s for 30 to 60 min. All experiments were conducted at room temperature (T=20 °C).

## Conflict of interest

The authors declare no conflict of interest.

1

## Supporting information

As a service to our authors and readers, this journal provides supporting information supplied by the authors. Such materials are peer reviewed and may be re‐organized for online delivery, but are not copy‐edited or typeset. Technical support issues arising from supporting information (other than missing files) should be addressed to the authors.

Supporting Information

## Data Availability

The data that support the findings of this study are available from the corresponding author upon reasonable request.
