# Supplementary material for: A Pitfall in Heavy Metal Separation with Amino‐modified Silica Adsorbents from Aqueous Solution: The Occurring pH Shift
Source: ChemistryOpen. 2022 Mar 10;11(5):e202200034. doi: 10.1002/open.202200034 (PMC9059311; doi:10.1002/open.202200034)
Supplement: Supplementary file 1 — Supporting Information [file OPEN-11-e202200034-s001.pdf]

ChemistryOpen

Supporting Information

A Pitfall in Heavy Metal Separation with Amino-modified Silica Adsorbents from Aqueous Solution: The Occurring pH Shift

Friederike Kriese,* Stephan Lassen,* and Helena Horn

Table of Contents

Results and Discussion 1
Calculation of Saturation Concentration of Metal Hydroxides at Different pH Values 1
Batch Mode Experiments 1
Continuous Mode Experiments 2
References 2

Results and Discussion

Calculation of Saturation Concentration of Metal Hydroxides at Different pH Values

The calculated saturation concentrations of $Ni(OH)_2$ and $Co(OH)_2$ $S_{Me(OH)2}$ at pH of 9 were computed by the following equation.[1]

$$\log S_{Me(OH)_2} = \log K_{L,Me(OH)_2} + 2 \cdot pK_w - 2 \cdot pH$$

The following parameters were used: pK_w = 14.0 (25 °C), pH = 9.0, $pK_{L,Co(OH)2}$ = 14.9, $pK_{L,Ni(OH)2}$ = 15.2 (25 °C). [1]

Batch Mode Experiments

Table S1: pH value in equilibrium state depending on adsorptive (Ni(II), Co(II), initial concentration (0, 30 or 3000 µmol L^{-1}), adsorbent (MonoA, TriA) and initial pH value pH_0 in discontinuous mode adsorption experiment (T = 20 °C)

Adsorptive Concentration / µmol L^{-1}	No Adsorptive 0	Ni(II) 30	Co(II) 30	Ni(II) 3000	Co(II) 3000
pH_0 = 6.0					
MonoA	9.8	9.8	9.9	7.6	7.8
TriA	9.5	9.6	9.6	7.5	7.1
pH_0 = 3.5					
MonoA	9.7	9.6	9.6	7.7	7.6
TriA	9.4	9.4	9.5	7.3	6.7
pH_0 = 2.2					
MonoA	6.7	6.9	7.0	7.2	7.2
TriA	8.0	8.2	8.2	6.4	6.5

Table S2: Removal Efficiency in equilibrium state depending on adsorptive (Ni(II), Co(II)), initial concentration (30 or 3000 µmol L^{-1}), adsorbent (MonoA, TriA) and initial pH value pH_0 in discontinuous mode adsorption experiment (T = 20 °C)

Removal Efficiency / %	Ni(II) 30	Co(II) 30	Ni(II) 3000	Co(II) 3000
$pH_{t=0}$ = 6.0				
MonoA	97.6	100	26.8	50.7
TriA	98.3	54.0	45.0	40.7
pH_0 = 3.5				
MonoA	94.0	100	31.3	26.8
TriA	93.4	56.7	45.2	35.7
pH_0 = 2.2				
MonoA	1.46	8.33	2.42	4.33
TriA	92.0	56.3	35.7	41.3

Continuous Mode Experiments

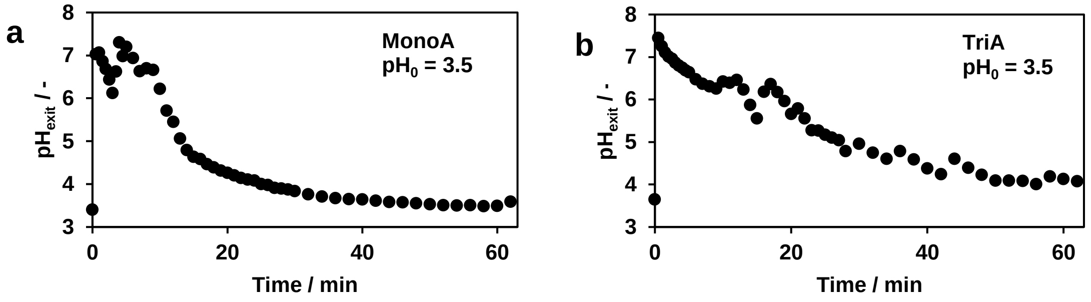

Figure S1: Exiting pH value pH_{exit} as a function of time (continuous mode) without adsorptive for MonoA (a) and TriA (b). Initial pH value of pH_0 = 3.5 at T = 20 °C.

References

[1] F. Scholz, H. Kahlert, *Chemical Equilibria in Analytical Chemistry*, Springer, Berlin, **2019**, p.115-127
